# Preserved micturition after intradetrusor onabotulinumtoxinA injection for treatment of neurogenic bladder dysfunction in Parkinson’s disease

**DOI:** 10.1186/s12894-016-0174-2

**Published:** 2016-09-05

**Authors:** Stephanie C. Knüpfer, Susanne A. Schneider, Mareike M. Averhoff, Carsten M. Naumann, Günther Deuschl, Klaus-Peter Jünemann, Moritz F. Hamann

**Affiliations:** 1 Department of Urology and Pediatric Urology, University Medical Centre Schleswig-Holstein, Arnold-Heller-Strasse 3, Campus Kiel, 24105 Germany; 2 Department of Neurology, University Medical Centre Schleswig-Holstein, Campus Kiel, Germany

**Keywords:** Parkinson’s disease, OnabotulinumtoxinA (OnabotA) injection, Neurogenic lower urinary tract dysfunction, International Consultation and Incontinence Questionnaire-Lower Urinary Tract Symptoms Quality of Life (ICIQ-UI)

## Abstract

**Background:**

To assess the efficacy and safety of intradetrusor onabotulinumtoxinA (OnabotA) injection treatment in patients with neurogenic lower urinary tract dysfunction (NLUTD), especially for patients with Parkinson disease (PD).

**Methods:**

PD patients refractory to oral antimuscarinic participated in an off-label use study and were evaluated prior and after 200 IU OnabotA injection into detrusor muscle, including trigone. Changes due to treatment were evaluated using bladder diaries, urodynamics, and questionnaires. Statistical analysis comprised Wilcoxon rank-sum test. Values are presented as mean ± standard deviation.

**Results:**

Ten PD patients (4 female and 6 male, mean age: 67.9 ± 5.36 years) with LUTD were enrolled. All patients tolerated the treatment. Bladder diary variables decreased significantly (*p* ≤ *0.011*) after OnabotA injection compared to variables prior injection. Desire to void and maximum bladder capacity increased significantly in urodynamics (*p* ≤ *0.05*). Maximum detrusor pressure during voiding phase normalised from 56.2 to 18.75 cm/H_2_0. Detrusor overactivity was less often detectable. All patients voided spontaneously. Mean post void residual (PVR) volume was 77.0 ± 119.78 mL postoperatively. No urinary retention or side effects have been observed during/after treatment. Mean follow-up time was 4 months (range of 1–12). 4 patients requested repeated injection after a mean period of 10 months between first and second injection.

**Conclusions:**

Our data confirm the efficacy and safety of 200 IU OnabotA injection in patients with neurogenic LUTD due to PD. The risk of urinary retention or high post-urinary residual volumes seems to be minor after OnabotA-injection. More research is needed with larger sample size to confirm the significance of these findings.

**Trial registration:**

Trial Number: ISRCTN 11857462, Registration Date 2016/10/08

## Background

Lower urinary tract dysfunction (LUTD) such as urinary urgency, frequency, and incontinence commonly occurs in neurological diseases, having a significant impact on quality of life, affecting emotional, social, sexual, occupational, and physical aspects of daily life [1]. Therapy of such neurogenic LUTD (NLUTD) is challenging, because all available treatment modalities (i.e. conservative, minimally invasive and surgical treatments) may fail or cause significant side effects.

Parkinson’s disease (PD) is the second most common neurodegenerative disease following Alzheimer’s disease, both associated with considerable socio-economic burden [2]. Epidemiological studies revealed that LUTD may be the most common non-motor manifestation affecting about 27–64 % of PD patients. Due to neurogenic detrusor overactivity, which challenges more the urinary storage phase than the voiding phase, those patients mainly suffer from urinary urgency, increased urinary frequency (both during the day-time and particularly at night-time) and incontinence.

Several factors may account for the prevalence and severity of LUTD among PD patients, including disease duration, gender, and urinary tract comorbidities such as benign prostate hyperplasia (BPH) and alterations in the LUT related to aging. However, to date a clear correlation between LUT symptoms (LUTS), disease duration, neurological impairment, and age in patients with PD has not been established. Thus, to determine presence and severity of LUTD in PD standard neuro-urological evaluation including urodynamic investigations and established questionnaires, such as the International Consultation on Incontinence Questionnaire (ICIQ) are essential to obtain a precise diagnosis of LUTD and to improve the choice and adjustment of treatments.

In general, the dopaminergic system plays an important role in physiological micturition [3, 4]. However, little is known about the mechanism inducing neurogenic bladder overactivity in this disease. Dopaminergic neurons project to the pontine micturition center [5]. There is increasing evidence that dopaminergic pathway influences micturition depending on the type of dopamine receptors that are activated [4]. It has been demonstrated in animals, that activation of D1-like dopamine receptors causes inhibition, whereas D2-like receptors are involved in facilitation of the micturition reflex [6].

In PD, the degeneration of dopaminergic neurons in the substantia nigra and the subsequent loss of striatal dopamine cause neurogenic detrusor overactivity, which might be attributed to the deactivation of the D1-mediated tonic inhibition [3, 7].

Besides dopaminergic agents, which have an effect on motor symptoms but only a slight effect on LUTD [8], antimuscarinics are generally used as a first-line treatment [9]. However, the treatment benefit is limited by central side effects (i.e. dry mouth, constipation, cognitive impairment), which occur in approximately 60 % of treated PD patients [3]. Moreover, simultaneous supplementation of antimuscarinics and PD medication is limited by the negative interaction [10]. Furthermore, antimuscarinics have yet not been evaluated in randomized multi-center trials, and should thus be prescribed with caution in PD patients.

Apart from deep brain stimulation, which seems to improve bladder capacity, to decrease detrusor overactivity, and to increase the volume at first desire to void [11] no second or third line therapy for LUTD in PD patients is approved to date. In most cases pads, intermittent self or indwelling catheters or even invasive surgical treatments (i.e. bladder augmentation or urinary diversions) remain when urinary frequency and subsequent urinary incontinence persists and/or patients become severely immobile. These options are related to significant long-term complications. Thus, enhanced treatment is urgently needed.

Intradetrusor OnabotA-injections have emerged as an effective, minimally invasive, well-tolerated and widely accepted treatment for refractory neurogenic detrusor overactivity incontinence [12]. Although the mechanism of action of OnabotA has not been clarified completely, it is assumed that OnabotA inhibits vesicular acetylcholine release into the neuromuscular junction and thus induces reversible denervation of extrafusal motor fibers, which weakens muscle contraction [13].

Recently, 100 IU intradetrusor OnabotA-injections were noted to effectively alleviate detrusor overactivity in patients with PD without causing urinary retention or increasing the post void residual (PVR) [14]. Drug treatment usually starts with low dosage and might be increased in the case of dissatisfied treatment success. According to the literature, there are four small studies exploring the effect of intradetrusor OnabotA injection for LUTD in patients suffering from PD (Table 1). To our knowledge intradetrusor 200 IU OnabotA injection has only been systematically assessed in a series of 4 female patients [15]. Thus, there is still a lack of data, which elucidate the impact of intradetrusor 200 IU OnabotA-injections in male patients as well as in a larger sample size with PD.

**Table 1:** Review of literature with comparison of previous and present studies of OnabotA-injections in PD

Reference	Sample size	Age [yrs]	Gender	Disease duration [yrs]	Dosage/sites	Injection localisation	Outcome measure	Result	Follow-up
Giannantoni et al. [15]	4	72–83	F (4)	4–12	200 IU/20	Intradetrusor incl. Trigone	UDI bladder diary Pressure flow QoL	Urinary frequency (day-/nighttime) decreased No urgency/urge incontinence ICIQ/UDI improved PVR increased no side effects	1/3 months 5 months Telephone interview
Giannantoni et al. [22]	8	66 ± 3	F (7) M (1)	N/A	100 IU/10	Intradetrusor	UDI bladder diary	Urinary frequency (day-/nighttime) decreased ICIQ/UDI improved PVR increased PVR 250 mL -- > ISC in 2 female patients	1/3/6 months
Anderson et al. [14]	20	71.5	F (8) M (12)	10.6	100 IU/10–20	Intradetrusor incl. Trigone	UDI bladder diary Pressure flow QoL (KHQ)	Urinary frequency (day-/nighttime) decreased PVR increased AUA Symptom score decreased	6 months
Kulaksizoglu et al. [21]	16	67.2 ± 5.1	F (10) M (6)	6	500 IU/30 Dysport®	Intradetrusor	bladder diary QoL (SEAPI)	Urinary frequency (day-/nighttime) decreased No incontinence in 27 % patients ICIQ VAS scale caregiver PVR increased no side effects	1 week 3/6/9/12 months
Present study	10	67.9 ± 5.3	F (4) M (6)	9.2 ± 8.2	200 IU/20	Intradetrusor incl. Trigone	UDI bladder diary QoL (ICIQ)	Urinary frequency (day-/nighttime) decreased UDI improved QoL (ICIQ) improved PVR increased no side effects	4 months

*yrs* years, *F* female, *M* male, *IU* international units, *ICIQ* international consultation on incontinence questionnaire, *UDI* urodynamic investigation, *PVR* post void residual, *ISC* internittend self-catheterization, *AUA* American urological association, *VAS* visual analog scale, *QoL* quality of life, *incl*. inclusive

The aim of the study was to investigate effectiveness and safety of intradetrusor 200 IU OnabotA injections in patients with LUTD due to PD. We hypothesized that 200 IU OnabotA injection would effectively alleviate the LUTD in the patient group and voluntary voiding would still be possible.

## Methods

Ten patients (4 female and 6 male, mean age: 67.9 ± 5.36 years) diagnosed with PD and LUTD refractory to at least two different types oral antimuscarinics were enrolled and participated in this off-label use study limited to 10 patients. All patients gave informed written consent. Study inclusion criteria were refractory LUTD with concomittant PD, as documented by a bladder diary, with urgency frequency syndrome and/or urgency incontinence refractory to antimuscarinics (i.e. Fesoterodinfumarat 4 mg/8 mg, Solifenacin succinat or Trospium) for at least 4 weeks. Patients were studied while on their usual drug regimens for PD, which included levodopa and dopaminergic agonists. One PD patient had deep brain stimulation of the subthalamic nucleus. No patient suffered from diabetes mellitus. Voluntary voiding was preserved in all patients. Patients were informed about the possibility of some form of catheterization if necessary, preferably intermittent self-catheterization (ISC) after OnabotA treatment. None of the patients was on anticoagulant therapy. Study exclusion criteria were unstable neurological disease, LUT malignancy, previous OnabotA treatment, untreated LUT obstruction, and missing informed consent.

Prior (visit 1) and in general 4 months postoperatively (visit 2), all patients underwent neuro-urological evaluation consisting of medical history, clinical examination, urine analysis, urinary tract ultrasound, urodynamic investigation, and urethrocystoscopy [16]. Urodynamics were performed according to good urodynamic practices as recommended by the International Continence Society (ICS) [17]. Patients were investigated in a sitting position. The bladder was filled with a room temperature mixture of 0.9 % NaCL solution and contrast medium. The clinical examination included digital rectal examination, vaginal inspection, and transrectal sonography. Urodynamics indicated in three patients a mild obstruction due to BPH (mean prostate volume: 49.5 ± 17.77 mL, PVR <50 % of the total bladder capacity), which disappeared after photo-selective laser vaporization of the prostate (PVP) prior to OnabotA-injection.

Quality of life (QOL) of patients was assessed using validated and highly recommended [18] International Consultation and Incontinence Questionnaire-Lower Urinary Tract Symptoms Quality of Life (ICIQ-LUT Sqol). This questionnaire contains 19 items on various aspects of QOL, which might be affected by leakage and another 3 items related to personal relationship. Total scores range from 19 to 76 points, with higher values indicating greater impact. The procedure was performed under general (or spinal, *n* = 3) anaesthesia.

### Injection technique

Intradetrusor OnabotA-injections were performed under cystoscopic guidance. A total of 200 IU OnabotA (Botox, Allergan Inc., Irvine, CA) diluted in 10 IU per/mL were injected using a flexible injection needle (BONEE Ch/Fr 05, 1.7 mm, Coloplast) into the detrusor muscle distributed among 16–17 submucosal/intradetrusor sites and 3–4 sites into the trigone due to the results of Abdel-Meguid et al. [19]. The injection was given gently and penetration of the detrusor muscle and thus injection into perivesical tissues was prevented. Currently, no other injection technique could demonstrate any significant advantage [20]. Thus, the standard intradetrusorial injection technique was used based on several published data [12, 29].

Postoperatively, in all patients bladder drainage (indwelling 16Ch Foley catheter) was maintained for at least 24 h to prevent complications (bladder bleeding, urethral and bladder neck edema). After the removal of the catheter and prior to patients discharge, spontaneous micturition and bladder emptying was verified based on PVR. Follow-up neuro-urological evaluation was done in general 4 month after OnabotA-injection.

### Statistical analysis

Statistical analyses were performed using IBM SPSS Statistics 19 for Windows (IBM™ Illinois, USA). Analyses were performed using Wilcoxon signed rank test for paired samples, due to the not normal distribution of the data tested by Kolmogorov-Smirnov test. For all statistical analysis a significance level of *p* < 0.05 was used. All values are presented as mean ± standard deviation (SD).

## Results

**Fig. 1:**
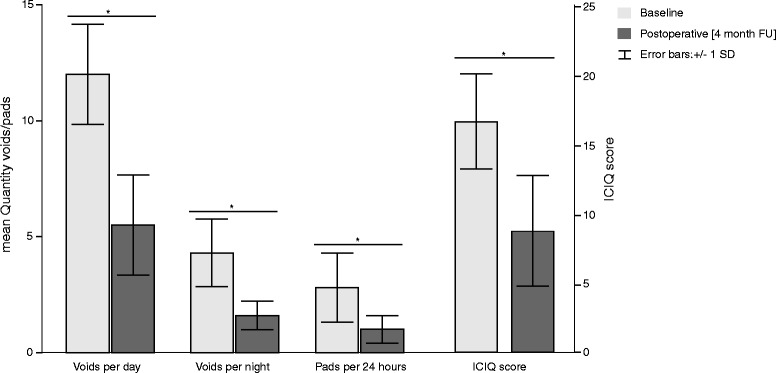
Three day bladder diary results regarding day-time and night-time frequency and pad consumption and International Consultation and Incontinence Questionnaire-Lower Urinary Tract Symptoms Quality of Life (ICIQ-LUT Sqol) in all patients with PD at baseline visit (*light grey bars*) and postoperative visit (4 month follow up (FU), *dark grey bars*). Data presented as mean ± standard deviation (SD). Asterisk indicates *p* ≤ 0.05. Significance level is 0.05

**Fig. 2:**
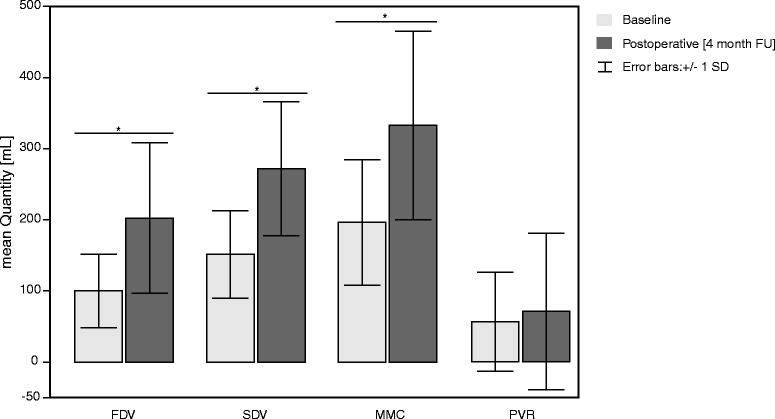
Volume at first desire to void (FDV), strong desire to void (SDV), maximal cystometric capacity (MMC), and post void residual (PVR) at baseline visit (*light grey bars*) and postoperative visit (4 month follow up (FU), *dark grey bars*). Data presented as mean ± standard deviation (SD). Asterisk indicates *p* ≤ 0.05. Significance level is 0.05

Ten patients (4 female and 6 male, mean age: 67.9 ± 5.36 years) diagnosed with PD (mean duration: 9.2 ± 8.2 years) and LUTD refractory to oral antimuscarinics participated in the study. The procedure was well tolerated. Epidemiological and clinical patients’ characteristics are summarized in Table 2. Preoperatively all ten patients complained about increased daytime (mean: 12.0 ± 3.39 times per day) and night-time (mean: 4.3 ± 2.32 times per night) urinary frequency, and a high pad consumption (mean: 2.8 ± 2.35 per day) due to urinary incontinence episode (Table 2). The mean ICIQ score was 16.63 ± 3.4 points (Table 2). All patients voided spontaneously, only 1 of 10 patients had a PVR greater than 150 mL. None of the patients performed the ISC. Preoperative urodynamic investigations showed detrusor overactivity in 9 of 10 patients, characterized by a low threshold desire to void, high detrusor pressure and decreased maximum cystometric capacity (MCC) (Table 3). Postoperatively, bladder diary data showed significant decreased urinary frequency (day-and nighttime; *p* = *0.005*, Fig. 1) and in pad use (*p* = *0.01*, Fig. 1) compared to the situation before OnabotA-injection, which is reflected in the significantly improved ICIQ score (*p* = *0.018*, Fig. 1). Overall, urodynamic investigations confirmed an improvement (Table 3). Mean MCC increased significantly from 196.2 ± 88.29 mL preoperatively to 332.6 ± 135.45 mL postoperatively (*p* = *0.005*, Fig. 2). Detrusor overactivity was observed on urodynamic in 9 patients before and in 2 patients after OnabotA-injection. Postoperatively, mean bladder volume increased significantly from 100 ± 51.51 mL to 202.38 ± 105.82 mL and from 151.3 ± 61.41 mL to 271.5 ± 94.07 mL (*p* ≤ *0.05*) at first desire to void (FDV) and strong desire to void (SDV), respectively (Fig. 2). In 8 patients the maximum detrusor pressure in the contraction period of micturition decreased significantly from a mean of 57.9 ± 33.1 cmH_2_O preoperatively to 18 ± 16.55 cmH_2_O postoperatively (*p* = *0.018*). No significant differences was found between the maximum flow rate (Qmax) before and after OnabotA injection (*p* = *0.212*). Although, PVR increased from 61.28 ± 75.91 mL to 77.0 ± 119.78 mL postoperatively, a significant difference was not observed (Fig. 2). In 2 patients PVR increased beyond the upper normal limit of 100 ml, however, they remained clinically asymptomatic. In all patients the micturition was preserved after OnabotA-injection. Patients were discharged from hospital 24 h after catheter removal and confirmation of spontaneous voiding. No form of catheterization was needed.

**Table 2:** Patients characteristics

Diagnosis	*n*	Sex	Age [yrs]	Disease duration [yrs]	ICIQ		Day time frequency		Night time frequency		Pad consumption	
					Visit 1	Visit 2	Visit 1	Visit 2	Visit 1	Visit 2	Visit 1	Visit 2
PD	10	4 F, 6 M	67.9 ± 5.36	9.2 ± 8.2	16.63 ± 3.40	8.75 ± 3.99*	12.72 ± 3.39	5.50 ± 3.4**	4.30 ± 2.32	1.6 ± 0.97**	2.8 ± 2.35	1 ± 0.94*

Total mean (±SD) values of age (years), disease duration (years), bladder diary parameters, and ICIQ score from 10 patients suffering from Parkinson disease (PD) at baseline visit (Visit 1) and postoperative visit (Visit 2)

*PD* Parkinson disease, *F* female, *M* male, *yrs* years, *ICIQ* international consultation on incontinence questionnaire, significance level: *p ≤ 0.05, **p = 0.005

**Table 3:** Urodynamic findings in the current study

Urodynamics	Mean ± SD Visit 1	Mean ± SD Visit 2	*p* Value
First Urge to Void (mL)	100 ± 51.51	202.38 ± 105.82	0.050
Strong Urge to Void (mL)	151.3 ± 61.41	271.5 ± 94.07	0.017
Maximal cystometric capacity (mL)	196.2 ± 88.29	332.6 ± 135.45	0.005
Maximum detrusor pressure during voiding phase [cm/H2O]	57.9 ± 33.1	18 ± 16.55	0.018
Bladder compliance [mL/cmH20]	18.65 ± 6.19	29.75 ± 28.79	0.123
Maximum flow rate [mL/s]	10.4 ± 3.14	13.03 ± 4.8	0.212
Voided volume [mL]	131.7 ± 96.56	246.8 ± 113.39	0.005
Post void residual [mL]	61.28 ± 75.91	77.0 ± 119.78	0.575

Total mean (±SD) values of urodynamic parameters from 10 patients suffering from Parkinson disease (PD) at baseline visit (Visit 1) and postoperative visit (Visit 2). Significance level of *t*-test is 0.05

During the 4-month’s follow up no adverse advent included urinary tract infection or haematuria occurred. 4 of 10 patients received a re-injection due to declining effect after a mean period of 9.75 ± 4.85 months. Postoperatively, the antiparkinson medication remained unchanged in all patients.

## Discussion

In line with previous studies [14, 15, 21, 22], we found in PD patients a significant improvement in urodynamic-, bladder diary parameters and consequently in the ICIQ score after 200 IU OnabotA injection with preserved micturition. The mean age of our patients was representative of the PD population.

The application of OnabotA injection in patients with NLUTD was pioneered by Schurch and colleagues [22]. After OnabotA injection (200 of 300 IU, Botox®, Allergan) in patients with spinal cord injury urodynamic-as well as bladder diary parameters improved significantly [23]. Since then, several studies, including randomized, placebo-controlled trials have approved the evidence of OnabotA injection in the treatment of neurogenic detrusor overactivity (NDO) using different study protocols [12, 24, 25].

Despite the fact that efficacy and safety of OnabotA injection has been studied in patients suffering from multiple sclerosis (MS) and spinal cord injury (SCI), there are only few data providing information on the impact of OnabotA injection on urodynamic-, bladder diary parameters, and ICIQ scores, including the specific dosage of 200 IU in PD patients. Currently, only 4 studies are available describing the effect of OnabotA injection in a PD population (Table 1).

Due to different study protocols used, the comparison of results from recent studies [14, 15, 21, 22] with our data is difficult. However, most parameters at the follow-up visits provided global improvement in urodynamic-and/or bladder diary parameters. With respect to decreased urinary frequency (day-and night time) results from the study by Anderson et al. [14] appear to be less pronounced than in the present study. Our data provide significant changes in the urinary frequency related to OnabotA-injection, which could be due to the elevated dosage of 200 IU. However, previous literature [22] also observed a significant improvement on urodynamic-and bladder diary parameters using 100 IU OnabotA-injection. Unfortunately, the latter findings mainly focused on female patients, and thus hamper a comparison with the present results.

Currently only two studies in PD patients [15, 21] are available describing the effects of an increased dosage exceeding 200 IU of intradetrusor OnabotA-injections. The first study, published by Giannantoni and colleagues [15] used OnabotA in a mixed population consisting of PD and multiple system atrophy. It demonstrated significant improvement in urodynamic-, bladder diary parameters as well as in the QoL. Our data confirm these effects in a considerably larger PD population. The second of these studies [21] used a different agent, Dysport ®, which may hamper an comparison due to particular pharmacodynamics.

Evidence concerning relevant PVR in patients treated with 200 IU OnabotA injection intravesically is controversial and sparse. Recently, published data showed an increased PVR and even urinary retention in patients with NLUTD, excluding PD [12, 26]. In contrast to these observations, White et al. [27] detected no PVR, when using 200 IU OnabotA-injections. Again, these controversial results might be attributed to the heterogeneity of patients’ cohorts with clinical symptoms of idiopathic or neurogenic detrusor overactivity. In our PD group PVR increased after the injection of 200 IU OnabotA. The increase was not significant and no form of catheterization was needed. Physiologically, this might be attributed to the fact, that only few PD patients demonstrate detrusor-sphincter dyssynergia [28] and thus voiding function remains unimpaired. On the other hand a dose dependent impact of OnabotA on detrusor contractility has to be taken into account. Although low dosage of OnabotA injection does not necessarily prevent the need for de novo ISC or even indwelling catheterization [22] the reduction of detrusor contractility in patients with NLUTD remains a relevant factor [12, 26]. Thus, independently of the dosage used, PD patients should be encouraged to learn ISC prior to OnabotA injection.

According to treatment technique, the injection into the bladder trigone is still under debate. The first reported injection techniques spared the trigone due to the potential complication of vesicourethral reflux (VUR) [29]. However, a recent prospective study evaluated the impact of trigonal OnabotA injection (300 IU) and observed no de novo VUR [19]. In addition, Lucioni et al. [30] observed no significant differences between the effects of sole trigonal and combined trigonal and intradetrusor OnabotA injection (300 IU). With regard to PD patients, the studies by Giannantoni [22], Anderson et al. [14] and the present study observed no treatment complication after trigonal OnabotA injection which might indicate a good eligibility of the technique in the selected patient group.

Impaired cognitive function is common in PD, affecting up to 30 % of patients, which might be aggravated by anaesthesia [31]. But to date, it is unclear how far general anaesthesia impairs cognitive performance of PD patients. In line with the literature [15, 22], we mainly performed OnabotA-injections under general anaesthesia. Giannantoni et al. [22] excluded patients with cognitive impairment. However, in none of our patients an extended distinctive cognitive impairment was noted within a period of up to 4 months postoperatively. In all treated patients orientation, memory, and attention was present. Noteworthy, Anderson [14] and Kulaksizoglu [21] performed OnabotA treatment under local anaesthesia with respect to comorbidities and possible interactions with other parkinsonian medications this approach seems to be favourable in PD patients.

To avoid obstructive problems studies by Giannantoni et al. [15, 22] focused on female cases: overall 12 cases included only one male. The present study investigates both sexes. In fact, three of our patients received a PVP 3 months prior to OnabotA injection due to a mild obstructive micturition which didn’t change PVR and urinary urgency significantly (mean change in PVR 28.4 (±52.7), ml *p* = *0.82*). This approach is in line with results of Kessler et al. that showed that TURP resolves urge symptoms in up to 70 % [32]. But, according to the non-significant change in PVR between mean values of preoperative and postoperative data, it is unlikely that only PVP is the cause of the global improvement in urodynamic-and/or bladder diary parameters. Especially the persistent urinary urgency after PVP in those patients are caused by urogenic pathogenesis rather than subvesical obstruction.

In the follow-up, patients demonstrated significant improvement for QoL. To evaluate the QoL we chose the short from of ICIQ-LUT Sqol questionnaire, recommended by the International Continence Society (ICS), to make the evaluation of QoL more practical, particularly with regard to existing tremor in the upper limb.

Although we demonstrated a preserved micturition after intradetrusor OnabotA injection (200 IU) for treatment of NLUTD in Parkinson’s disease this study is limited by the small sample size, which is probably the cause of the high SD in some outcome parameters. Furthermore, it cannot be ruled out that comorbidities and their specific therapies do not affect in a limited extent the results of the bladder function. Nevertheless, our results, based on the largest published series are promising. All patients experienced significant benefit from OnabotA injection, which justify further prospective placebo-controlled investigations in this special patient group.

## Conclusions

Patients with PD have a high prevalence of LUTD and most commonly suffer from storage but not voiding symptoms due to NDO. Although the exact mechanism of how OnabotA injection modulates NDO is not clear, OnabotA injection (200 IU) is an effective and safe treatment method in patients with PD due to the significantly improved of urodynamic-, bladder diary parameters, and QoL due to ICIQ. In contrast to the common apprehension based on observation in other neurogenic bladder dysfunctions, the risk of urinary retention or increased post void residual bladder volume seems to be a minimal in PD patients.

However, the number of investigated patients is low with high inter study heterogeneity and there is a lack of randomized controlled trials. Thus, well-designed, adequately powered studies are needed before more widespread use of OnabotA injection (200 IU) for neurogenic LUTD can be recommended.

### Ethical and dissemination

This trial was performed in accordance with the World Medical Association Declaration of Helsinki [33], the guidelines for Good Clinical Practice [34]. This study was approved by the local ethics committee (Ethikkommision Kiel) and all patients gave written informed consent according to the Helsinki II declaration. Handling of all personal data will strictly comply with the Good Clinical Practice [34].

## Abbreviation

BPH: Benign prostate hyperplasia
FDV: First desire to void
FSFI: Female sexual function index
ICIQ: International consultation on incontinence questionnaire
ICIQ-LUT Sqol: International consultation and incontinence questionnaire-lower urinary tract symptoms quality of life
ICS: International continence society
IIEF: International index of erectile function
ISC: Intermittent self-catheterization
IU: International units
LUTD: Lower urinary tract dysfunction
LUTS: Lower urinary tract syndrome
MCC: Maximum cystometric capacity
MS: Multiple sclerosis
NDO: Neurogenic detrusor overactivity
OnabotA: Onabotulinumtoxin
PD: Parkinson’s disease
PVP: Photo-selective laser vaporization of the prostate
PVR: Post void residual
Qmax: Maximum flow rate
QOL: Quality of life
SCI: Spinal cord injury
SD: Standard deviation
SDV: Strong desire to void
